# The role of health volunteers in promoting people´s knowledge, attitude and practice towards COVID-19 in Iran: strategies and challenges

**DOI:** 10.11604/pamj.2021.39.87.29946

**Published:** 2021-05-28

**Authors:** Javad Nazari, Saeed Amini

**Affiliations:** 1 Department of Pediatrics, Medical School, Arak University of Medical Sciences, Arak, Iran,; 2 Department of Health Management, School of Health, Khomein University of Medical Sciences, Khomein, Iran

**Keywords:** Health volunteers, self-care, COVID-19

## To the editors of the Pan African Medical Journal

More than a year has passed since the COVID-19 outbreak. During this year, we have witnessed various waves of this virus along with high morbidity and mortality [1]. These waves indicate that reliance on official and governmental forces and facilities to some extent can help control the disease. Despite the measures that have been taken to control COVID-19, every arisen wave of the disease reflects the fact that people´s knowledge, attitude and practice have not been sufficiently improved to prevent another new waves [2]. At the same time, we are witnessing the exhaustion and fatigue of health care staff, and if we do not want to see other waves of the disease, it is necessary to institutionalize self-care behaviors among the people. To achieve this goal, it can be very helpful to use health providers similar to the people they live with, that are named peer groups.

According to the Alma-Ata declaration, people have right and duty to participate in the planning and implementation of their health care, individually and collectively [3]. In this regard, the Iranian health system has launched the project of health volunteers in 1990. Despite the valuable actions of the forces active in this project, but in the opinion of the authors of the present article who have provided and leaded healthcare services for many years, this program has been weak and with minimal activity and recruitment in the recent years. Now that we are witnessing many problems and challenges in controlling COVID-19, the role of health volunteers is becoming clearer and it is necessary to review this network.

Health volunteers in Iran are divided into groups of family, student, collegian, neighborhood, and organizations volunteers. Health volunteers learn training methods, ways to communicate with people, and self- care courses at health centers, and participate in health center planning sessions to address health problems. Given the high prevalence of COVID-19 and considering that there is no specific vaccine or drug to prevent and treat this disease, the most important way to prevent and control the disease is to follow the hygienic principles, including 6 key steps of regular hand washing for 20 seconds, using face masks, prevent touching the eyes, nose and mouth, prevent shaking hands and kissing, resting at home when sick, throwing used tissue paper into trash with lid. It is necessary for all people to receive the necessary training to prevent and control the disease. Through the volunteers the training is transferred in chain among the community and eventually to the entire population. The cost-effectiveness of the health volunteers actions has been confirmed in various studies and there is no debate about to be or not to be the volunteers [4,5]. Health volunteer services increase public satisfaction and give opportunity to health system devote their time and resources on other important issues [6].

### Identifying, educating and supporting heath volunteers

In order to health volunteers be able to help the health system to achieve its goals, it is necessary for the heath deputy of medical universities to implement measures and programs to improve their capabilities. For this, at the first, it is necessary to educate the educators of health volunteers themselves regarding the services that health volunteers provide. These educators include health center experts in the fields of health education, health advocacy, health providers, and supporting forces. In the second step, various organizations and institutions including schools, universities, NGOs, and other private and public organizations should introduce their health volunteers to health centers. Lastly, these volunteers should be educated by the stated health experts. In addition to holding in-person sessions to train health volunteers, utilization of the infrastructures of virtual training is useful. For example, educational messages may be transferred to student health volunteers through social media or organizations´ health volunteers can be targeted through official letters, office portals, office automation and social networks.

### Evaluation health volunteers´ implementation

The activities of medical universities and health networks should not be limited to attracting and educating the volunteers. It is important to monitor and evaluate the actions taken so that we can examine the extent to which the goals have been achieved. Indicators that can be used for this purpose include the number and percentage of attracted households, neighborhoods, students, offices, organizations, factories and workshops health volunteers that have been recruited and trained. Also, assessment of the level of health literacy promotion of different groups of people and their ability to apply health recommendations, performing health behaviors in preventing the disease, staying at home, not traveling, etc. are other indicators of the program evaluation.

### Supporting health volunteers

Health volunteers in exchange of voluntary and non for profit services they provide to the people should not be ignored from the supporting services of medical universities and the health centers. Providing personal protective equipment for them such as masks and disinfectants, financial and non- financial incentives, honoring ceremonies, and celebrations are some of these supporting programs.

### Conclusion

Successful implementation of the health volunteers program requires macro-level planning and strengthening the implementation infrastructures. Certainly, the existence of people's forces that are interested in and committed to the health of society and then holding training courses for them, alongside the official health care system, can lead to the promotion of people's self- care. Because of decentralized implementation, health volunteers can target the real needs of local people, as a result lead to change in the knowledge, attitude and practice of the people and boost people self-confidence.
